# Effective prevention of post-dural puncture headache with insertion of an intrathecal catheter in parturients: a retrospective study and meta-analysis

**DOI:** 10.1186/s44158-023-00107-5

**Published:** 2023-07-20

**Authors:** F. Creazzola, M. Aversano, F. Prencipe, R. Barelli, P. Pasqualetti, I. Simonelli, M. G. Frigo

**Affiliations:** 1 Obstetric Anaesthesia, San Camillo Forlanini Hospital, Circonvallazione Gianicolense 87, 00152 Rome, RM Italy; 2 Obstetric Anaesthesia and Intensive Care Departmental Unit, Fatebenefratelli Gemelli Isola - Isola Tiberina Hospital, Via Di Ponte Quattro Capi 39, Rome, RM 00186 Italy; 3 Anaesthesia and Intensive Care Unit, Sant’Eugenio Hospital, Piazzale Dell’Umanesimo 10, 00144 Rome, RM Italy; 4 Service of Medical Statistics and Information Technology, Fatebenefratelli Foundation for Health Research and Education, Via Di Ponte Quattro Capi 39, 00186 Rome, RM Italy

**Keywords:** Labour epidural analgesiaPost-dural puncture headache, Accidental dural puncture, Intrathecal catheter

## Abstract

**Background:**

Accidental dural puncture is a common complication of labour analgesia. It can trigger post-dural puncture headache, with associated morbidity and increased costs. Intrathecal catheter placement is a prophylactic procedure which can reduce incidence and severity of post-dural puncture headache.

**Methods:**

We conducted a retrospective single-centred study to define incidence and risk factors of accidental dural puncture and post-dural puncture headache in an obstetric population. We also evaluated effectiveness of intrathecal catheter placement compared to epidural catheter replacement in reducing incidence of post-dural puncture headache. We then conducted a systematic review and meta-analysis which included all studies comparing intrathecal catheter placement to epidural catheter replacement in obstetric patients with accidental dural puncture assessing the outcome of reduced incidence of post-dural puncture headache as a dichotomous variable.

**Results:**

Accidental dural puncture had an incidence of 0.25% (60 cases). Of these, 66% developed post-dural puncture headache. A total of 77% (47/60) of patients with accidental dural puncture were treated with an intrathecal catheter placement, while 23% (13/60) had an epidural catheter replacement. Incidence of post-dural puncture headache was lower in the intrathecal catheter group (spinal 26/47, 60.5% epidural 11/13, 84.6%), although not reaching statistical significance (*RR* 0.71, *CI* 95%: 0.51–1.00; *p* = 0.049). The meta-analysis revealed that intrathecal catheter placement significantly reduced incidence of post-dural puncture headache compared to epidural catheter replacement (pooled *RR* 0.81, 95% *CI* 0.72–0.91, *p* < 0.001).

**Conclusions:**

Intrathecal catheter placement is a promising measure to prevent post-dural puncture headache, especially if followed by a pain management protocol and a continuous saline infusion.

## Introduction

Epidural analgesia affords more effective pain relief in labour than non-epidural techniques [1]. Accidental dural puncture (ADP) is one of its most common complications, with an incidence between 0.19 and 3.6%, depending on materials used and operator’s experience [2]. Post-dural puncture headache (PDPH) develops in up to 80% of patients following ADP with 16- to 18-gauge epidural needles [3] and can be associated with significant maternal distress, increased hospital length of stay, costs, and increased workload for the anesthesiologist [4]. According to the International Headache Society, PDPH is defined as ‘headache occurring within 5 days of a lumbar puncture, caused by cerebrospinal fluid (CSF) leakage through the dural puncture, usually accompanied by neck stiffness and/or subjective hearing symptoms. It remits spontaneously within 2 weeks or after sealing of the leak with autologous epidural patch’ [5]. According to the available guidelines [6–8], the gold standard treatment for PDPH is performance of an epidural blood patch (EBP). However, such invasive technique carries several risks and shows higher efficacy if performed 48 h from dural puncture [8], while prophylactic measures against development of PDPH are performed immediately after dural puncture. After ADP, adequate analgesia must be guaranteed, and anaesthesiologist must choose between epidural catheter replacement (ECR) or inserting an intrathecal catheter (ITC). In the first case, a new epidural catheter is positioned with the same loss of resistance technique (LOR) in the same or different intervertebral space with an additional risk of accidental dural puncture [1, 8, 9]. The advantages of an ITC placement are the reduction of incidence and severity of PDPH and the need for therapeutic blood patch [9–12], by stopping the CSF leak. Besides, it guarantees a rapid onset, high quality, and predictable labour analgesia [1] or anaesthesia if a caesarean section is required [11, 13].

Potential risks include infection, inadvertent local anaesthetic intoxication, and development of high blocks. Especially if used for a short period of time, ITC is associated with a very low rate of infection [14]. If adequate protocols are used, risks of local anaesthetic toxicity due to misuse of the catheter can be largely reduced [15]. Major concerns regarding ECR are a 9–10% risk of a second ADP [15, 16]. In our centre all the procedures have been managed by expert anaesthesiologists in obstetric. The primary endpoint of this retrospective study was to define incidence of ADP, of consequent PDPH and related risk factors during epidural or combined spino-epidural analgesia in an obstetric population. The secondary outcome was to evaluate effectiveness, in terms of reduction of PDPH and related symptoms, of a management protocol of ADP centred on ITC placement.

A systematic review of the literature and meta-analysis was then performed, which included all studies comparing ITC placement to ECR in obstetric patients with ADP to prevent PDPH. If the review confirms the benefits of ITCs, their widespread use may reduce postnatal distress of mothers, improve maternal-infant bonding, and reduce healthcare costs. However, if the benefits of ITCs do not exist, it will enable anaesthetists to avoid a procedure that has inherent, potentially serious risks [1].

## Methods

Our retrospective study was conducted in the Obstetrics Unit of the San Giovanni Calibita ‘Fatebenefratelli’ Hospital, with 3131 births and a percentage of neuraxial blockade performed on obstetric patients in labour of 92% for the year 2019. All parturients who underwent neuraxial blockade for labour between January 2010 and March 2018 and who developed ADP were included. ADP is managed following a structured protocol, which includes the placement of an intrathecal catheter used to administer intrathecal analgesia (or anaesthesia, if required). After delivery has occurred, the ITC is connected to an infusion pump and maintained for 36 h. This group of patients was compared with another group managed by an ECR due to the impossibility of place of the catheter in the subarachnoid space or by personal choice of the operator. ECR has been performed by repeating the procedure, with the same technique (LOR), placing the catheter in the same intervertebral space or in a different one. All data regarding ADP and PDPH are prospectively included in a dedicated database.

Demographic data and others regarding PDPH and associated symptoms were recorded (see Tables 1 and 2). Complications of treatment for PDPH were noted. All parturients were visited daily by an anaesthetist until hospital discharge. All personal information was de-identified, and medical records were analysed anonymously to protect patient privacy.

**Table 1:**
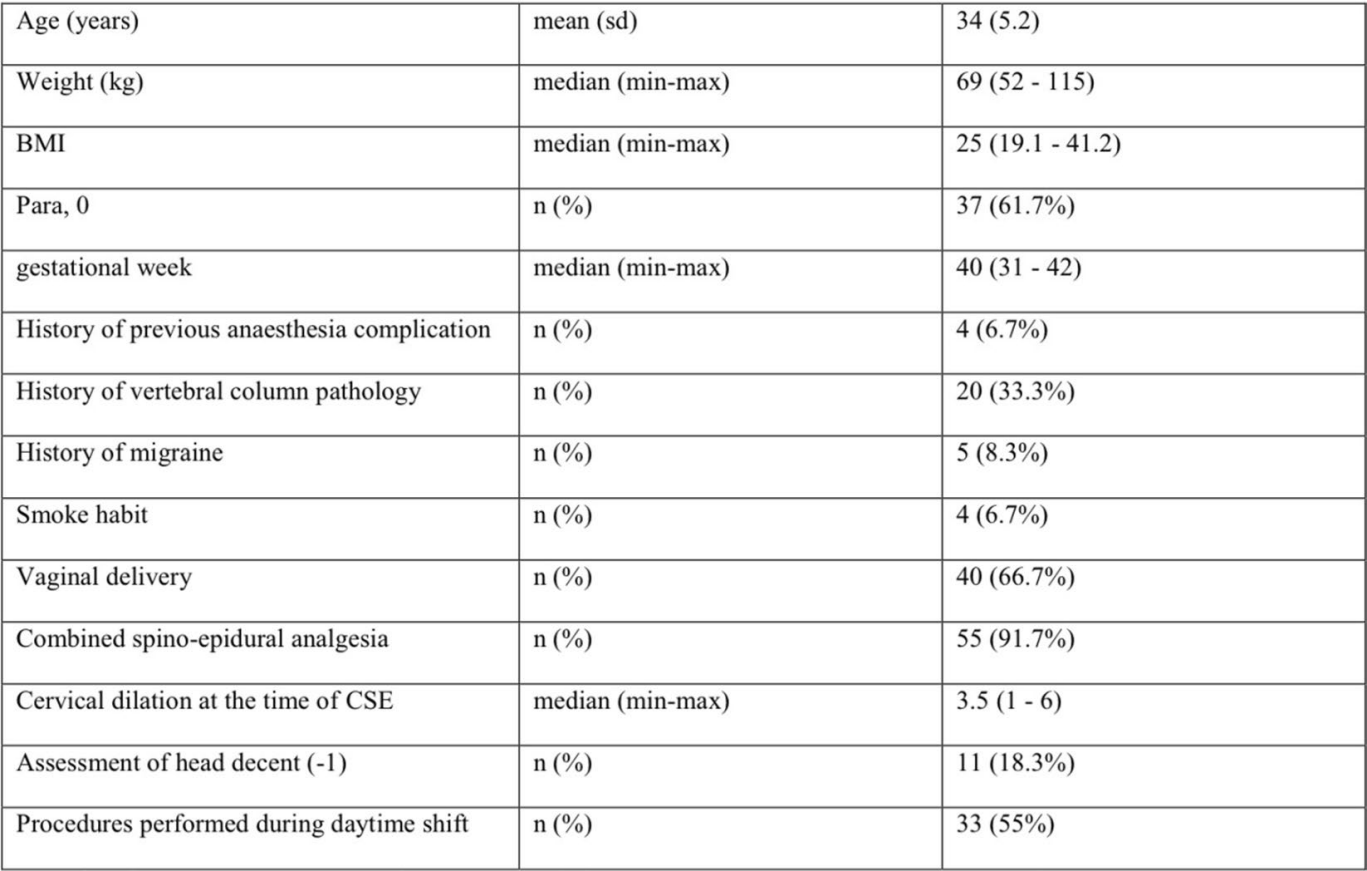
Demographic data for all patients who developed ADP

**Table 2:**
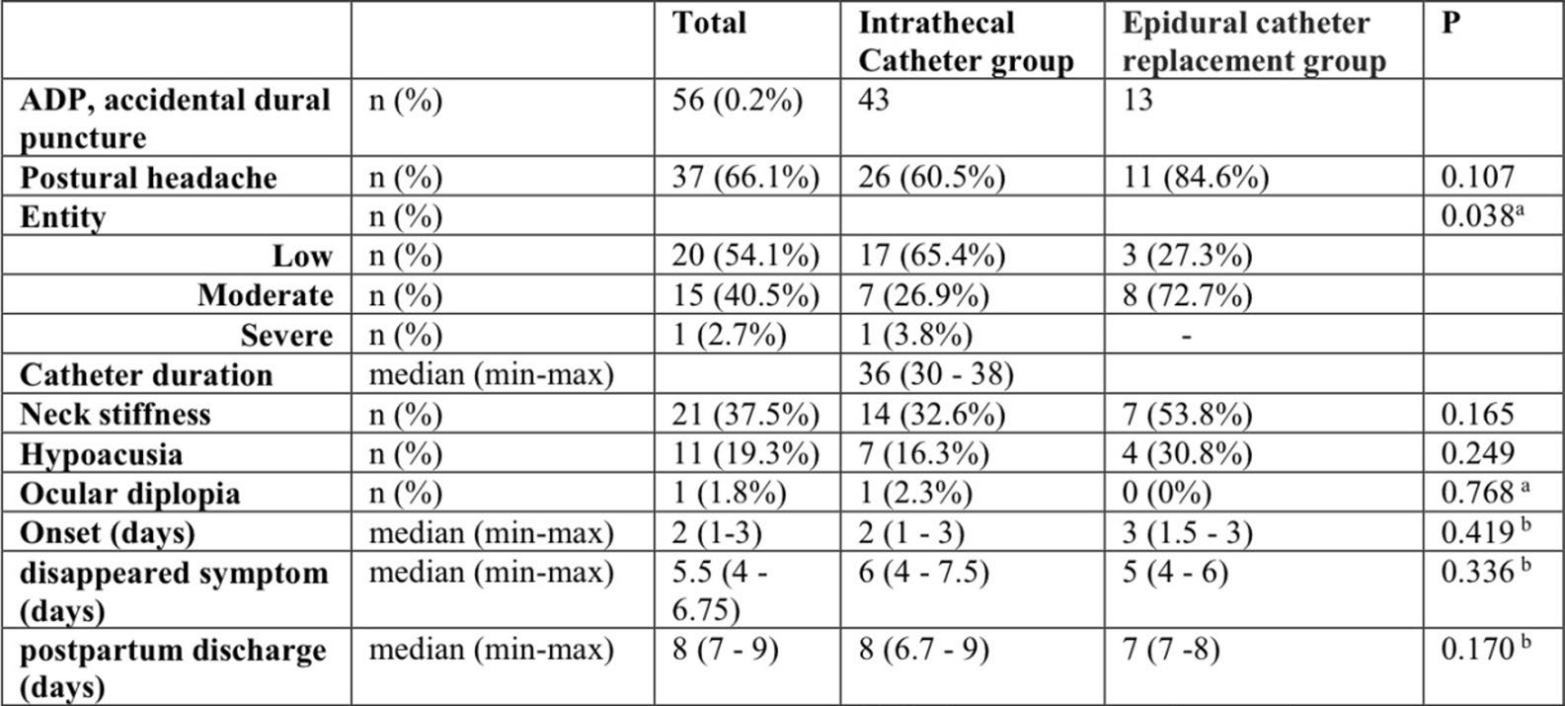
PDPH incidence, severity and associated symptoms in the ITC and ECR groups

Exclusion criteria were patients who developed PDPH after spinal anaesthesia or after a week or more since performance of epidural or combined spinal-epidural (CSE) technique. Approval for conduction of the study was obtained by the local ethical committee. All parturients received informed consent, both written and oral, which included both explanation of the technique used during labour and the protocol management of PDPH.

For the systematic review and meta-analysis, PRISMA guidelines were followed. Studies were included according to the following PICOS questions: all types of studies (excluded case reports and case series) which compared efficacy of ITC placement (intervention) to ECR (control) in reducing incidence or severity of PDPH after ADP (outcome) were included. Participants were women experiencing an ADP during an attempted insertion of epidural catheter in labour (population). Studies considering either immediate or delayed removal of ITC were both included. The primary outcome measure was incidence of PDPH, described as a dichotomous variable. Any mode of delivery was considered for inclusion. Articles published as abstracts were included. No limits were applied for language. Articles published up to March 2018 were included. The search was applied to PubMed, Embase, and Web of Science databases.

**Fig. 1:**
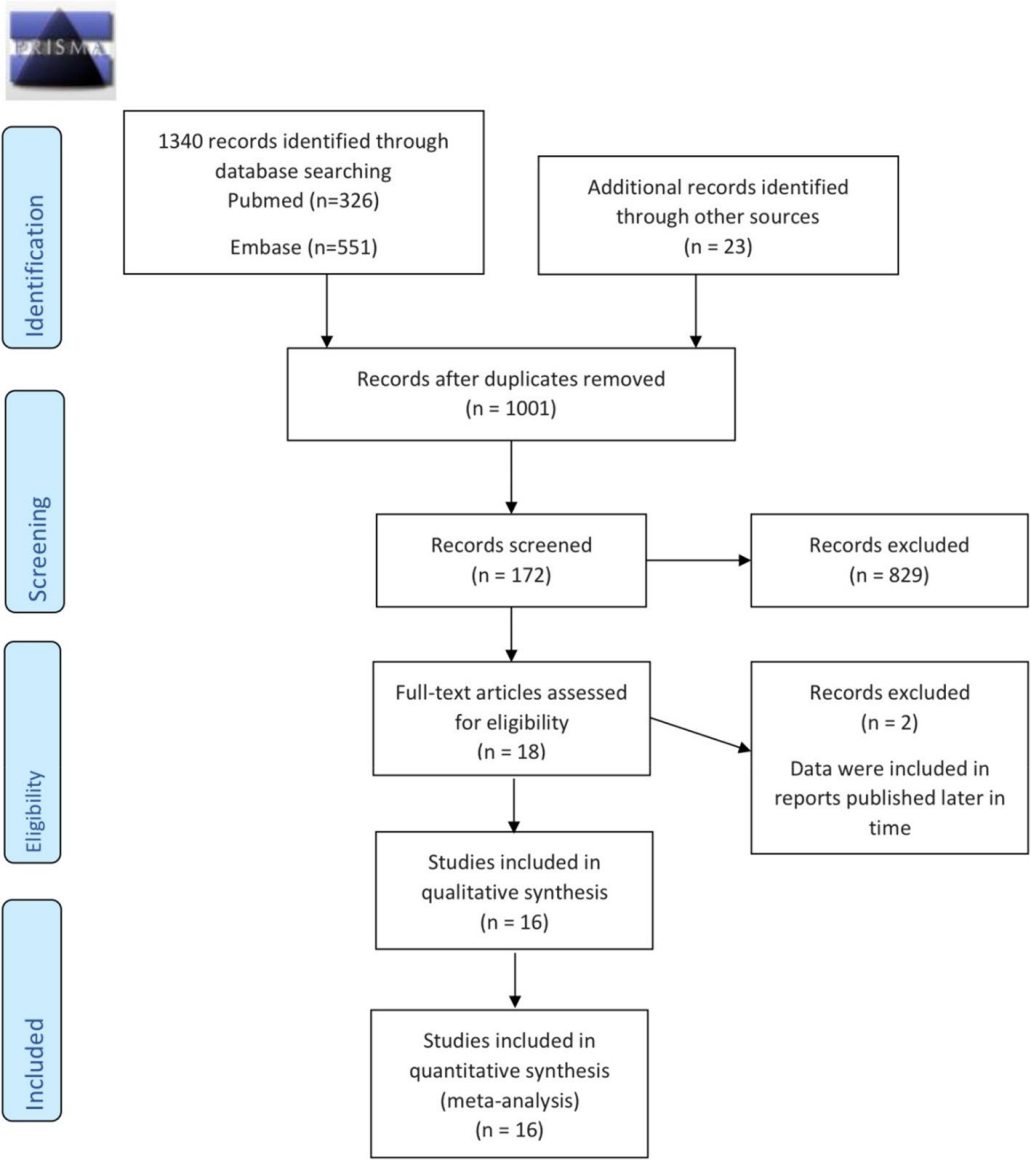
Flow diagram of the selection process of the included trials and specific reason for exclusion in the meta-analysis

The following key words were searched both as controlled descriptors (such as MeSH terms) and as unstructured terms, in each database: ‘accidental dural puncture’ OR ‘unintentional dural puncture’ OR ‘inadvertent dural puncture’ AND ‘intrathecal catheter’ OR ‘epidural catheter replacement. Two authors (F. C. and R. B.) independently examined all the potential studies selected from the search strategy against the inclusion criteria. All disagreements were resolved either through discussion or consulting a third author. For multiple publications from the same trial or same patients’ sample, we considered only one dataset. The process of study selection is described in the PRISMA flow diagram (Fig. 1). Two review authors (F. C. and R. B.) extracted the data independently from the included studies by collecting them in an Excel sheet. We resolved any differences through discussion. A third reviewer verified the quality of the data extraction (I. S.). Overall, we extracted first author’s name, published year, country, number of patients, study design, type of delivery, outcome (incidence of PDPH), ITC protocol, and criteria for assignment to treatment group. For the dichotomous outcome, incidence of PDPH, we extracted the number of participants with the event. Where reported, we directly extracted the incidence value. If data regarding methodology was absent, the first author was contacted. Trials were only included if sufficient information was available. No missing data imputation was done. Two review authors (M. G. F. and M. A.) independently assessed the methodological study quality with the Newcastle–Ottawa scale for cohort studies. The following domains were assessed: selection, comparability, and outcome/exposure. For each item, one star was given if considered of high quality. Any disagreements were resolved through discussion.

### Statistical analysis

Continuous data were represented in terms of mean (standard deviation, SD) or median (minimum maximum).

Categorial data were represented in terms of frequency (*n*) or percentage (%). The nonparametric Mann–Whitney test was used for the difference between ITC placement and ECR, being a continuous variable. Association with categorial variables was tested applying the chi-square or the Fisher test, where necessary. A *p*-value < 0.05 was considered statistically significant. Statistical analysis was performed with the software SPSS.

For the meta-analysis, risk ratio (RR) was used to compare PDPH treatment with an ITC versus treatment with ECR. The estimated effect was presented with 95% confidence interval (95% CI). The primary outcome was combined from the individual studies in a meta-analysis to provide a pooled effect estimate applying a random effects (RE) model. Heterogeneity was evaluated by visual inspection of a forest plot and measured by the Higgins index I [2, 17]; the values were interpreted following the guide suggested in the Cochrane Handbook [18]. A sensitivity analysis was performed excluding possible outlying data. Data were analysed with Review Manager (RevMan; Copenhagen: The Nordic Cochrane Centre, The Cochrane Collaboration, 2008) for meta-analysis. *p*-values < 0.05 were regarded as statistically significant. Publication bias was assessed both visually with a funnel plot and formally with the Harbord test.

In agreement with a previous study [10], we performed a subgroup analysis including studies which clearly documented that ITC removal was delayed for at least 24 h, in order to verify if delayed removal is associated with larger reduction of PDPH compared to immediate removal of ITC. With the aim of testing if infusion of saline in the ITC for at least 24 h is associated with reduced PDPH, we also performed a subgroup analysis including the studies in which such procedure was clearly documented.

## Results

Between 2010 and 2018, 24,050 women gave birth in our obstetric unit with labour analgesia or anaesthesia. ADP with a Tuohy needle had an incidence of 0.24% (60 cases). Of these, 66% developed PDPH, consistently with previous series [11]. Seventy-seven precent (47/60) of patients with ADP were treated with an intrathecal catheter inserted (ITC group), while 23% (13/60) had an epidural catheter replaced (ECR group). None of the patients recruited was treated with EBP. Table 1 shows relevant demographic and clinical data. Incidence of ADP did not increase in out-of-hour shifts, nor in case of vaginal delivery compared to caesarean section. Table 2 shows data comparing the ITC group and the ECR group. Incidence of headache was lower in the ITC group compared to the ERC group, although not reaching statistical significance (*RR* 0.71, *CI* 95%: 0.51–1.00; *p* = 0.049).

In 13 cases, the ADP management protocol (hence positioning of ITC) was not possible; in 4 of them, diagnosis of ADP took place after the end of the procedure; in 3 cases, the catheter was removed because of inadequate analgesia/anaesthesia; in 6 cases, causes were unknown. In 93% of cases, the operator realised occurrence of ADP during catheter insertion. Overall, adherence of clinicians to the ADP management protocol with ITC placement was 90%. Therapeutic EBP was never performed.

In our systematic review, after selection process, 24 studies were included [3, 4, 9–12, 15, 16, 19–33] in addition to unpublished data derived from our retrospective study, providing data on 1093 parturients with PDPH treated with an ITC and 968 treated with ECR. Data regarding study size, PICOS questions, and treatment protocols are described in Tables 3 and 4. The outcome measure was the risk of PDPH in all studies. In more than half of them, the outcome of therapeutic EBP was also considered. The latter was not considered in our meta-analysis, as such procedure is hardly ever performed in our obstetric unit, and we do not consider it as first-choice treatment.

**Table 3:**
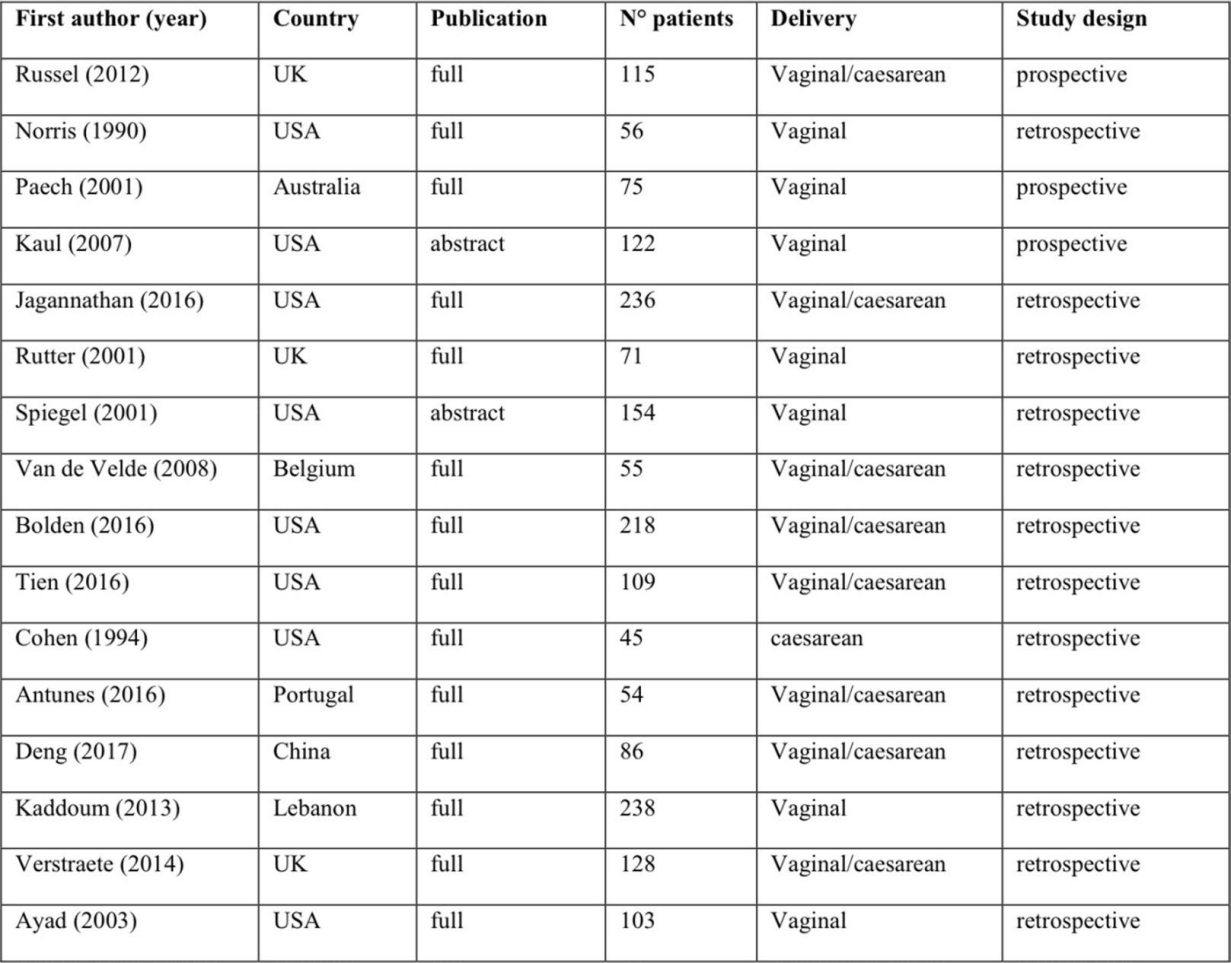
Characteristics of the 16 studies included in the meta-analysis

**Table 4:**
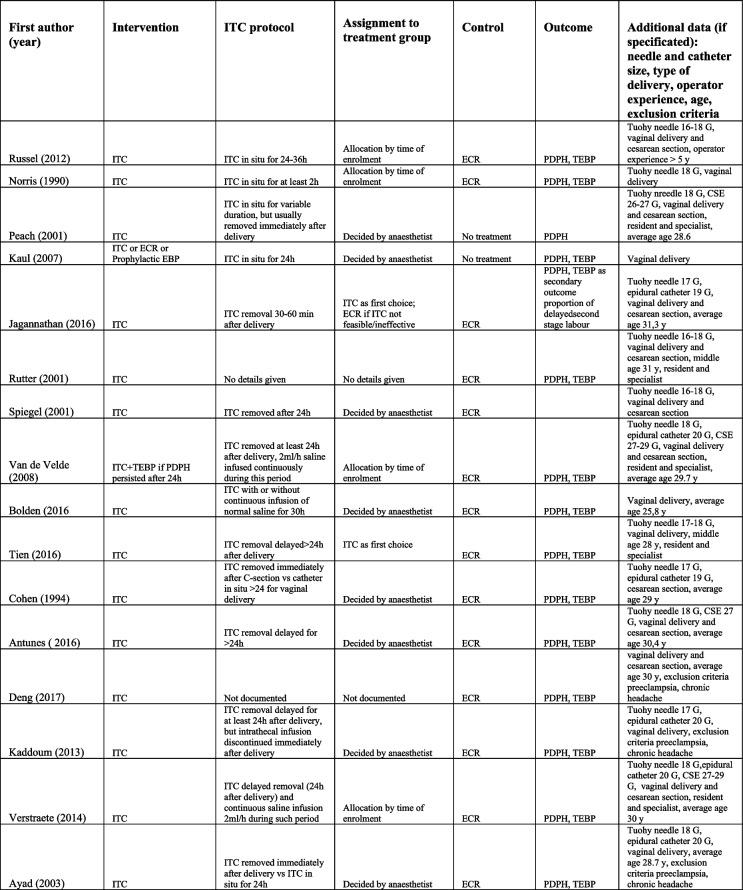
PICOS questions and intervention details of the studies included in the meta-analysis

Data regarding methodological study quality are reported in Table 5. To be noted that being the outcome a subjective one, its assessment is prone to a high risk of bias, unless a blinded outcome assessor is used, which was not documented in any of the cohort studies analysed. Cohort representativity was considered inadequate for studies who had exclusion criteria which limited population characteristics (e.g. only vaginal delivery included). Confounding factors considered were age, body mass index (BMI), mode of delivery, ITC protocol (if present and characteristics), and conservative management protocol. Only one study adjusted odd ratio (OR) for confounders [23].

**Table 5:**
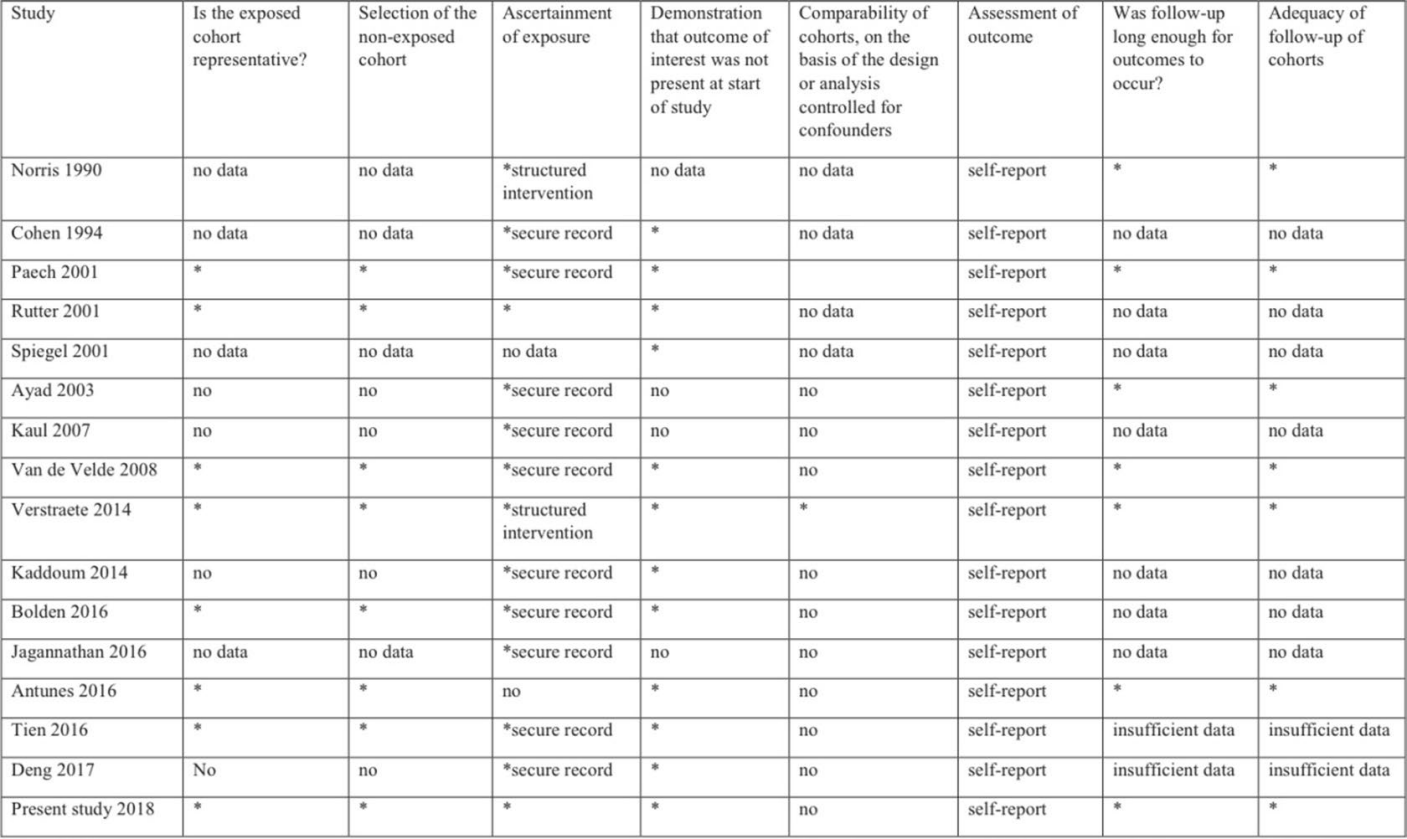
Risk of bias, Newcastle-Ottawa quality assessment scale for cohort studies

**Fig. 2:**
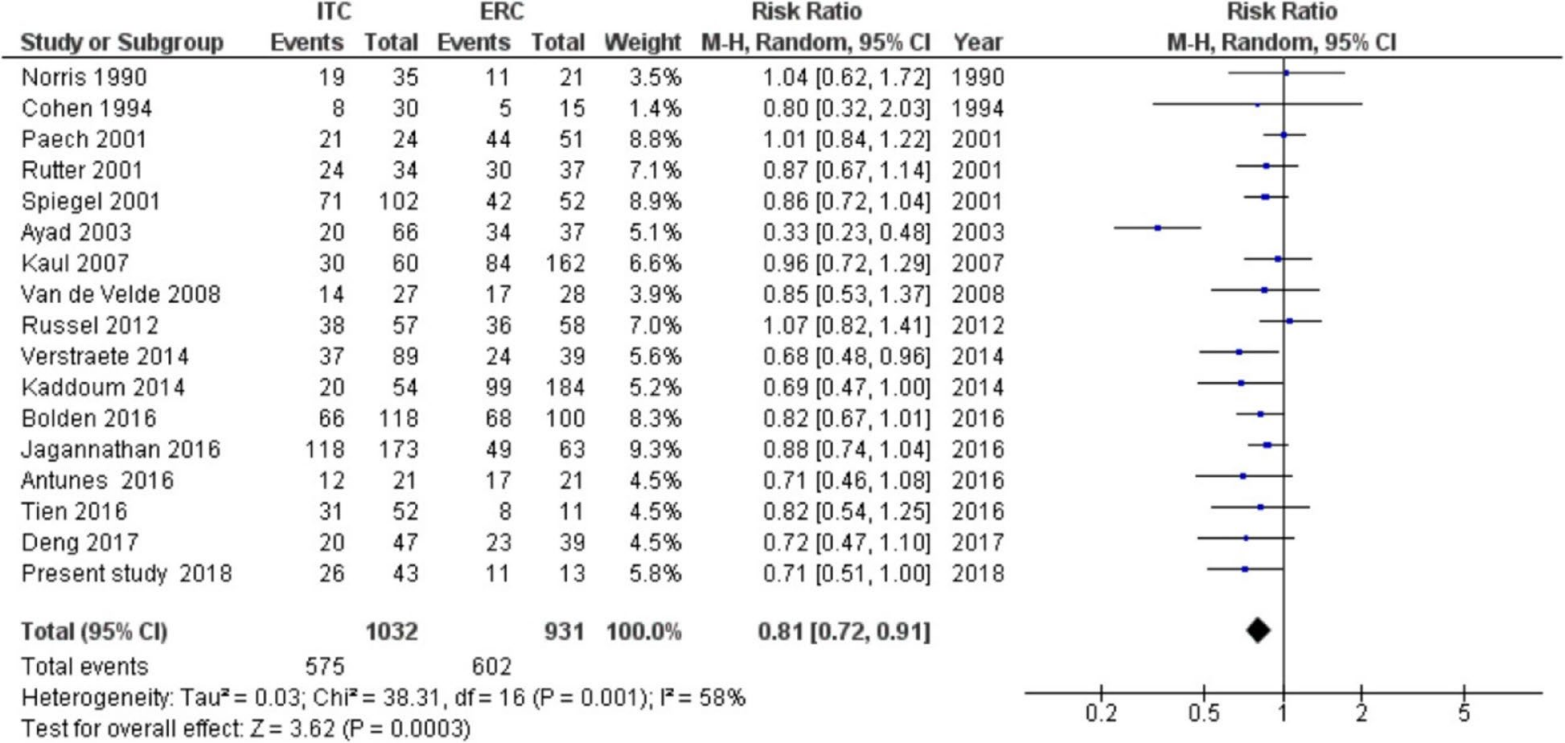
Forest plot of risk ratio (RR) of post-dural puncture headache ITC vs ERC

**Fig. 3:**
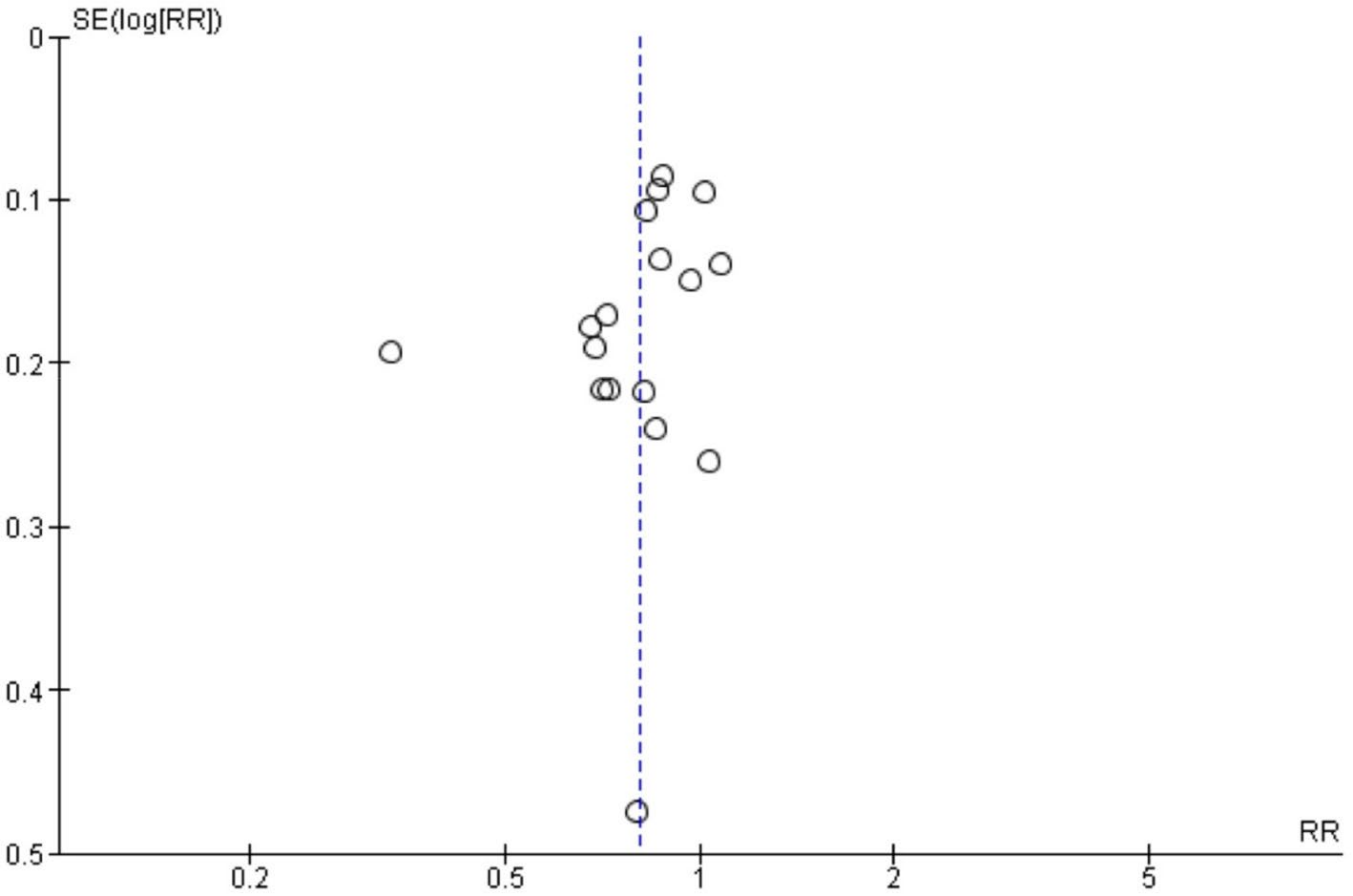
Funnel plot of studies included in the meta-analysis as a test for publication bias

**Fig. 4:**
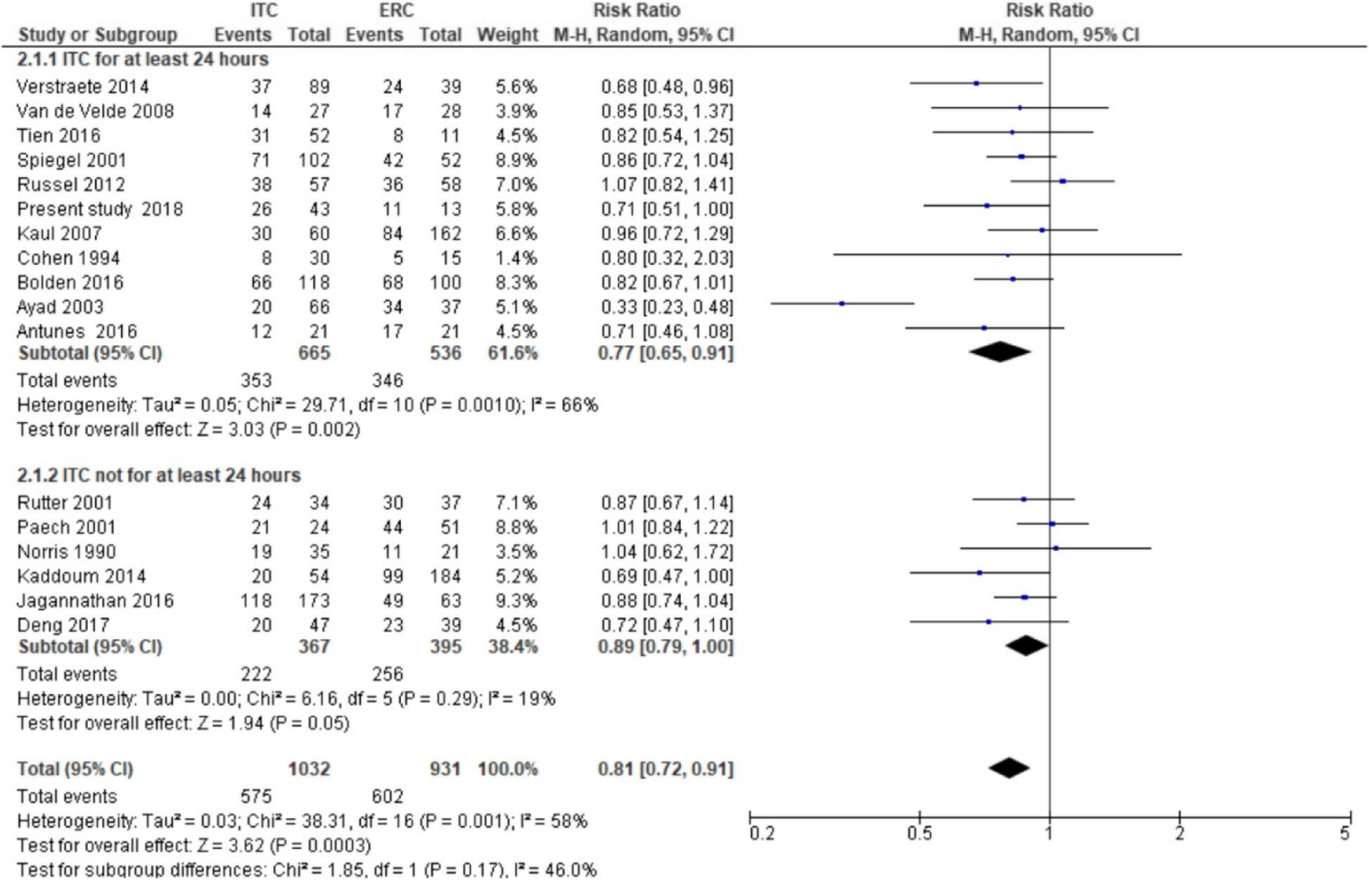
Subgroup analysis (delayed vs immediate ITC removal) forest plot

The pooled RR for PDPH was 0.81 (95% *CI* 0.72–0.91, *p* < 0.001) (Fig. 2). Heterogeneity was moderate and significant (*I*^2^ = 58.2%, *p* = 0.001). Observing the forest plot, one study [9] reported a result that was in the same direction of the other results but much lower. The funnel plot (Fig. 3) did not show evident asymmetry; this was confirmed by the results of Harbord test, bias =  − 1.62 (*SE* = 0.94, *p* = 0.106). A sensitivity analysis was performed to investigate heterogeneity excluding an outlying study [9]. The exclusion of the latter decreased the heterogeneity, *I*^2^ = 0% (*p* = 0.54), and confirmed the results of the main analysis: the risk of PDPH after treatment with an ITC was lower than that after treatment with an ERC (*RR* = 0.87, 95% *CI* 0.81–0.93, *p* < 0.001). When performing a subgroup analysis of studies with delayed ITC removal (Fig. 4), the pooled RR was significant (*RR* = 0.77, 95% *CI* 0.65–0.91; *p* = 0.002), but heterogeneity was substantial (*I*^2^ = 66%, *p* = 0.001). The pooled RR obtained from the remaining six studies was coherent with the result in the previous group, indicating a reduction of risk of PDPH after ITC compared with ERC placement (*RR* = 0.89, 95% *CI* 0.79–1.00; *p* = 0.05). Heterogeneity was not significant (*I*^2^ = 19%, *p* = 0.29). The test for subgroup differences indicated that there was no statistically significant subgroup effect (*p* = 0.17, analysis not presented), suggesting that the ITC removal delayed for at least 24 h does not modify the effect of ITC in comparison to ERC. However, the analysis may not be able to detect subgroup differences because of a smaller number of trials and participants in the immediate ITC removal subgroup compared to the ITC delayed removal subgroup.

Only four studies stated that infusion of saline in the ITC lasted for at least 24 h. The pooled result confirmed the result of the main analysis, *RR* = 0.77 (95% *CI* 0.67–0.90; *p* = 0.001), with no evidence of a significant heterogeneity (*I*^2^ = 0%, *p* = 0.73).

## Discussion

Data from our retrospective study showed an incidence of 0.24% of ADP, of which 66% developed PDPH, consistently with previous studies [3, 15, 34]. There was a lower incidence of PDPH in parturients treated with an ITC compared to a ECR, although not reaching statistical significance (*p* = 0.049). Data from our meta-analysis showed a statistically significant reduction in PDPH in the ITC group: pooled *RR* 0.823 (95% *CI* 0.72–0.89, *p*< 0.001). Such results, coherent with a recent similar study [26], may be explained by the fact that many single-centred studies — including ours — were underpowered, as a small difference in incidence needs larger sample size to be identified. When assessing clinical heterogenicity of data, several parameters varied among studies included in the meta-analysis, such as anaesthetist experience, mode of delivery, and epidural needle size. Some studies [10, 20, 23] showed that patients with delayed ITC removal (> 24 h) had a lower incidence of PDPH compared to the immediate ITC removal and the ECR groups. However, the subgroup analysis did not show a significant subgroup effect, possibly due to a small sample size. Creation of a hole in the meningeal membranes with a Tuohy needle determines leakage of CSF. This causes cerebral hypotension with traction on intracranial structures and compensatory vasodilation, which take to PDPH symptoms. It is reasonable that a normal saline bolus performed immediately after dural puncture can replace the volume leaked and reduce symptoms [35]. Only one study assessed the effect of a saline bolus in ITC compared to ITC without saline bolus, finding significant reduction in necessity of EBP in the first group [11].

As a single-centred retrospective study, possible limitations are the relatively small number of cases and the prolonged period of data collection. Although there was a high adherence to a predefined protocol regarding both analgesia/anaesthesia during labour and for the further 36 h, outcomes may have been influenced by clinical decisions. All the same, considering the low frequency of the event ADP, currently, retrospective studies are an important source of data for this topic. Among limitations of our meta-analysis, the authors consider the possibility of selection bias having influenced the results. Moreover, as pain management differed among studies and was often clinically determined rather than protocol based; modifications of clinical practice over time and among single clinicians might have influenced outcome. As very few data on complications were available among studies, no relevant conclusions can be drawn regarding side effects of ITC placement. Several alternatives to EBP and conservative treatments have been proposed as peripheral nerve blocks, such as sphenopalatine ganglion block (SPGP), greater occipital nerve block (GONB), and lesser occipital nerve block (LONB) [36].

## Conclusions

Our retrospective study and meta-analysis both indicate that ITC placement after ADP is a promising measure to prevent PDPH and associated symptoms, especially if followed by a pain management protocol and by a continuous normal saline infusion. PDPH affects a high number of parturients in the world and measures which can reduce its costs, and negative effects should be evaluated via further studies. Considering the numerous variables regarding the intervention considered, additional prospective randomised studies with predefined protocols regarding pain management, continuous infusion of normal saline, and ITC removal time are necessary.

## Data Availability

The datasets used and/or analysed during the current study are available from the corresponding author on reasonable request.
